# Transcriptomic meta-analysis to identify potential antifungal targets in *Candida albicans*

**DOI:** 10.1186/s12866-024-03213-8

**Published:** 2024-02-27

**Authors:** Zeinab Abdelmoghis Hefny, Boyang Ji, Ibrahim E. Elsemman, Jens Nielsen, Patrick Van Dijck

**Affiliations:** 1 Laboratory of Molecular Cell Biology, Department of Biology, Katholieke Universiteit Leuven, Kasteelpark Arenberg 31, Leuven, B-3001 Belgium; 2 BioInnovation Institute, Ole Maaløes Vej 3, Copenhagen, DK2200 Denmark; 3 Department of Information Systems, Faculty of Computers and Information, Assiut University, Assiut, 2071515 Egypt; 4 Department of Life Sciences, Chalmers University of Technology, Kemivägen 10, SE41296, Gothenburg, SE41296 Sweden

**Keywords:** *Candida albicans*, High-throughput sequencing, Differentially expressed genes, GO enrichment, Antifungal targets

## Abstract

**Background:**

*Candida albicans* is a fungal pathogen causing human infections. Here we investigated differential gene expression patterns and functional enrichment in *C. albicans* strains grown under different conditions.

**Methods:**

A systematic GEO database search identified 239 “*Candida albicans*” datasets, of which 14 were selected after rigorous criteria application. Retrieval of raw sequencing data from the ENA database was accompanied by essential metadata extraction from dataset descriptions and original articles. Pre-processing via the tailored nf-core pipeline for *C. albicans* involved alignment, gene/transcript quantification, and diverse quality control measures. Quality assessment via PCA and DESeq2 identified significant genes (FDR < = 0.05, log2-fold change > = 1 or <= -1), while topGO conducted GO term enrichment analysis. Exclusions were made based on data quality and strain relevance, resulting in the selection of seven datasets from the *SC5314* strain background for in-depth investigation.

**Results:**

The meta-analysis of seven selected studies unveiled a substantial number of genes exhibiting significant up-regulation (24,689) and down-regulation (18,074). These differentially expressed genes were further categorized into 2,497 significantly up-regulated and 2,573 significantly down-regulated Gene Ontology (GO) IDs. GO term enrichment analysis clustered these terms into distinct groups, providing insights into the functional implications. Three target gene lists were compiled based on previous studies, focusing on central metabolism, ion homeostasis, and pathogenicity. Frequency analysis revealed genes with higher occurrence within the identified GO clusters, suggesting their potential as antifungal targets. Notably, the genes *TPS2, TPS1, RIM21, PRA1, SAP4*, and *SAP6* exhibited higher frequencies within the clusters. Through frequency analysis within the GO clusters, several key genes emerged as potential targets for antifungal therapies. These include *RSP5, GLC7, SOD2, SOD5, SOD1, SOD6, SOD4, SOD3*, and *RIM101* which exhibited higher occurrence within the identified clusters.

**Conclusion:**

This comprehensive study significantly advances our understanding of the dynamic nature of gene expression in *C. albicans*. The identification of genes with enhanced potential as antifungal drug targets underpins their value for future interventions. The highlighted genes, including *TPS2, TPS1, RIM21, PRA1, SAP4, SAP6, RSP5, GLC7, SOD2, SOD5, SOD1, SOD6, SOD4, SOD3*, and *RIM101*, hold promise for the development of targeted antifungal therapies.

**Supplementary Information:**

The online version contains supplementary material available at 10.1186/s12866-024-03213-8.

## Background

Every year, 150 million people are infected with fungal pathogens which results in almost 1.7 million deaths yearly [1, 2]. A significant number of these infections are caused by *Candida* species, with *C. albicans* ranking as the fourth most prevalent cause of hospital-acquired bloodstream infections [3, 4]. In 2009, another *Candida* species, *C. auris*, was described for the first time and now, this species is causing infections everywhere in the world [5]. A major problem with this species is that most isolates are resistant to fluconazole and a large percentage of the isolates is also resistant towards echinocandins and polyenes [6, 7]. In many cases multidrug resistance is observed.

*Candida albicans*, an opportunistic human fungal pathogen, is frequently found in the human microbiome as a commensal in the gastrointestinal tract, oral cavity, and vaginal tract [8, 9]. However, this typical harmless species has the capacity to cause several types of infections depending on the host niche. It can cause mucosal infections in immune-competent persons, with vaginal infections as a typical example, but it can also cause systemic infections in immune-compromised patients, where different organs will be colonized, resulting to the death of the patient in many cases [3, 10–13]. The number of infections caused by *C. albicans* is also increasing because of the advancement of medical care in hospitals. More and more patients receive various types of implants, such as catheters, valves, hips, that are all suitable substrates for these fungal cells to attach to and to form a biofilm. The extracellular matrix produced by biofilm cells is known to sequester antifungal drugs resulting in tolerance towards these drugs. This barrier can even be further increased in multispecies biofilms [14–19]. Further, the increase in the use of broad-spectrum antibiotics is also increasing the incidence of fungal infections [11, 20, 21]. Finally, the increase in patients with diabetes or those receiving transplants also have a higher chance of getting a fungal infection [22].

*C. albicans* is a species that exhibits a great degree of adaptability, as it can grow in a variety of environmental conditions with varying nutrition availability, or differences in temperature, pH, osmolarity, and oxygen availability [23]. This flexibility is partly caused by the pleiotrophic morphologies that this species can grow in. Apart from growing as a budding yeast, they can also grow in the form of pseudohyphae and true hyphae, the morphology mostly associated with an infection [3, 24]. Hyphal cells can infiltrate tissues and cause damage [25–28] and they are important for the development of biofilms [29–34]. Several virulence factors are also associated with the hyphal morphology. These include the expression of different adhesin proteins, hydrolytic enzymes and the production of the candidalysin toxin, which can intercalate in the host cell membrane [8, 35–37].

Apart from understanding virulence factors, recognizing crucial predisposing elements for candidiasis development is also essential. Talapko et al. [38] reviewed factors like neutropenia, immunosuppression, diabetes, age, as well as those linked to patient care such as extended antimicrobial therapy, prolonged hospital stays, catheter use, and surgeries.

Candidiasis treatment options are limited to five chemical groups - polyenes, echinocandins, azoles, pyrimidine analogues, and allylamines, of which the first three are mainly used to treat systemic infections [39]. Azoles target ergosterol biosynthesis, polyenes bind to sterols in the membrane and echinocandins target cell wall biosynthesis. A major problem with these drugs, apart from being either fungistatic or toxic, is the rapid development of drug resistance. This is mainly a problem for the azoles and echinocandins [40–42] and this results in high mortality rates which can go up to 60% for *C. albicans* [43]. Bhattacharya et al. and Srivastava et al. [44, 45] reviewed the molecular mechanisms associated with antifungal resistance, including overexpression of transporters, alterations in cell wall/ergosterol production, and mutations in regulatory transcription factors. Further investigation into these mechanisms might aid in identifying resistant strains, discovering fresh drug targets, and restraining drug resistance progression.

The growing need for new antifungal agents stems from increased infections caused by resistant fungi and emerging strains, resulting in recent approvals of new antifungal drugs in 2021 and 2022 [2, 46, 47]. Novel antifungal drugs that are active on *Candida* sp. are fosmanogepix (interferes with mannoprotein production in the cell wall by inhibiting Gwt1), ibrexafungerp and rezafungin (a triterpenoid and novel echinocandin that both inhibit the β-(1,3)-glucan biosynthesis) and oteseconazole (novel tetrazole inhibiting ergosterol biosynthesis and used to treat vaginal infections) [48–50].

A major problem for the development of novel antifungal drugs is the fact that both fungi and humans are eukaryotes and phylogenetically seen, fungi are closely related to animals. This makes it difficult to identify enzymes or metabolic pathways that are specific for fungi and where inhibitors would not affect host cells. Whereas in the past, only essential proteins were targeted, now there is also more effort to target virulence factors, that are not necessarily essential for growth, but that are essential for virulence. There may be less pressure to develop resistance against such drugs [51].

The rapid advancements of omics technologies have resulted in a strong increase in the abundance of genomic data in public repositories. The use of “omics” techniques to study the biology of *C. albicans* has been significantly increased by the sequencing and annotation of the common laboratory strain, *SC5314* [52–54]. Key features in *C. albicans* are being examined using a variety of post-genomic approaches, such as comparative genomics, transcriptional profiling, and the construction of a full gene deletion collection [55].

For various biological purposes, large volumes of transcriptomic data have been generated using microarray or RNA sequencing technologies and are publicly available. Despite the biological and technical variability among samples in a given study, and the differences between studies, it is possible to systematically assemble and integrate the available gene expression data with the available meta-information on the different experimental conditions and workflows, in order to overcome unwanted variation as well as to increase the statistical power [56]. Such RNA-seq meta-analysis enables the generation of more complete data sets, the identification of individual research biases or shortcomings, the collection of the most reliable data, and the discovery of new trends and relationships [57].

In recent years, the integration of high-throughput sequencing and bioinformatics analysis data have revolutionized our understanding of genetic regulation, particularly in the context of *C. albians* infections. These approaches have enabled the identification of differentially expressed genes (DEGs) and the exploration of affected biological processes, molecular functions, and cellular components [58, 59]. Moreover, the utilization of Gene Ontology (GO) terms has proven invaluable in annotating gene product functions and predicting phenotypic outcomes [56, 60, 61]. Building on this progress, our work aims to further elucidate the distinct molecular characteristics exhibited by the identified GO terms under normal versus environmental stress conditions. By clustering these terms, we aimed to uncover a set of genes sensitive to various stress conditions, thereby identifying potential antifungal targets in *C. albicans*.

In this study, we compiled and analysed a comprehensive RNA-seq gene expression dataset through a meta-analysis approach. We integrated transcriptomics data from seven studies to extract the gene expression information under different conditions. This allowed us to reveal the presence of specific genes with high frequency within the up and down-regulated gene ontology clusters. Notably, these key genes involved in central metabolism, ion homeostasis, and pathogenicity emerged as potential targets for anti-fungal interventions.

Overall, our study deciphers the molecular mechanisms of *C. albicans* and identifies promising targets for the development of novel antifungal strategies by integrating gene expression information from multiple studies.

## Methods

### Data retrieval and pre-processing

**Fig. 1:**
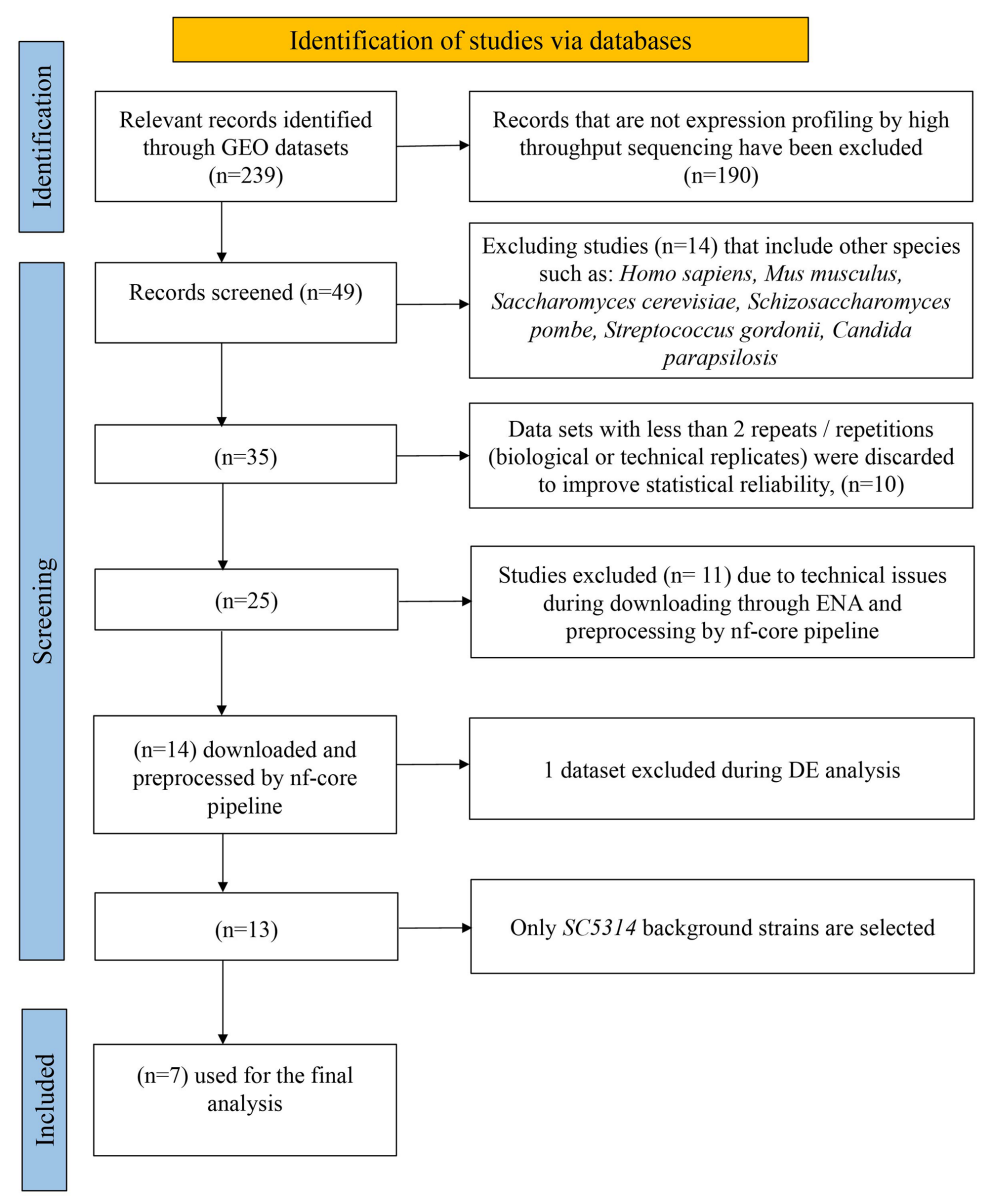
Selection Process of Relevant GEO Datasets for Analysis. This flow chart outlines the systematic process of dataset selection from the GEO repository that we used to perform the comprehensive analysis. Initially, 239 records were identified with the keyword ‘*Candida albicans*’. Excluding studies not employing high-throughput sequencing narrowed down the dataset to 190. A further 14 studies involving species other than *C. albicans* were eliminated. To ensure statistical robustness, 10 datasets with fewer than 2 repeats were discarded. Technical issues during data downloading and pre-processing led to the exclusion of 11 studies. Among the processed datasets (*n* = 14), one was excluded during differential expression analysis. The final selection focused solely on *SC5314* background strains, resulting in 7 datasets utilized for Gene Ontology (GO) Enrichment analysis. This streamlined selection process ensures the quality and relevance of datasets for the subsequent comprehensive analysis

A comprehensive search was conducted on the Gene Expression Omnibus (GEO) database [62] using the keyword “*Candida albicans*” resulting in the identification of 239 datasets. Following a rigorous selection process as shown in Figs. 1 and 14 datasets were retained for downstream analysis. Raw sequencing data for these selected datasets were downloaded from the European Nucleotide Archive (ENA) database [63] according to the accession numbers. Corresponding meta-information was extracted from the description of the data sets and the original articles, including genotype and treatment conditions, sample preparation and sequencing information.

The nf-core pipeline [64] was employed for pre-processing the raw data with configurations for *C. albicans*. The reference genome obtained from EnsemblFungi was converted to a GTF file and provided as input to the pipeline. Quality control (QC) reports and read counts were generated by the pipeline. In brief, the workflow processes raw data from FastQ inputs with FastQC [65], reads are then aligned with STAR [66], producing gene counts (featureCounts, StringTie) or transcripts (Salmon, tximport) and conducting comprehensive quality control on the results (RSeQC, Qualimap, dupRadar, Preseq, edgeR, MultiQC).

### Quality assessment and differential expression analysis

To assess the quality of the pre-processed data, principal component analysis (PCA) was performed using prompt function in the R environment with the log2-transformed counts as input. Subsequently, differentially expressed genes were identified using the DESeq2 package [67]. The obtained *p*-values are corrected for multiple testing using the Benjamini and Hochberg method using default setting in DESeq2. Genes were considered statistically significant if they exhibited a false discovery rate (FDR) of < = 0.05 and a log2-fold change > = 1 or <= -1. The GO term enrichment analysis was performed using topGO [68, 69]. GSE55819 [70] was excluded from further analysis due to low quality results. Additionally, GSE38298 [71], involving clinical isolates, and GSE103674 [72], focusing on white-opaque strains, were excluded from subsequent analyses. Moreover, GSE37682 [73], GSE75124 [74], GSE86540 [75], and GSE45141 [76] datasets were excluded as they utilized strains derived from the *SC5314* background (*CAF4-2, BWP17, SN152*). Consequently, the following datasets (GSE87832, GSE102039, GSE64659, GSE99902, GSE73409, GSE99767, GSE49310), all based on *SC5314* strain background, were chosen for further investigation. The details of these seven studies are provided in Table 1.

**Table 1:** Summary of the 7 selected RNA-seq studies used for the analysis

Reference	Study ID	Accession No.	No. of Conditions	No. of Replicates
[79]	5	GSE87832	4	2
[83]	7	GSE102039	4	2
[81]	8	GSE64659	2	3
[82]	9	GSE99902	10	6
[80]	12	GSE73409	8	5 or 3
[77]	13	GSE99767	12	4
[78]	14	GSE49310	6 × 4	4

## Results

### Study-specific clustering of gene expression patterns unveiled by principal component analysis (PCA)

The seven selected studies provide a comprehensive insight into *C. albicans*’ responses to a range of stimuli, including nutrient fluctuations, growth conditions, stressors, and pH variations (Additional Table 1**)**. Through rigorous RNA-seq analyses, these investigations unveil how gene expression patterns change under different conditions, shedding light on the intricate molecular mechanisms underlying *C. albicans*’ adaptability.

**Fig. 2:**
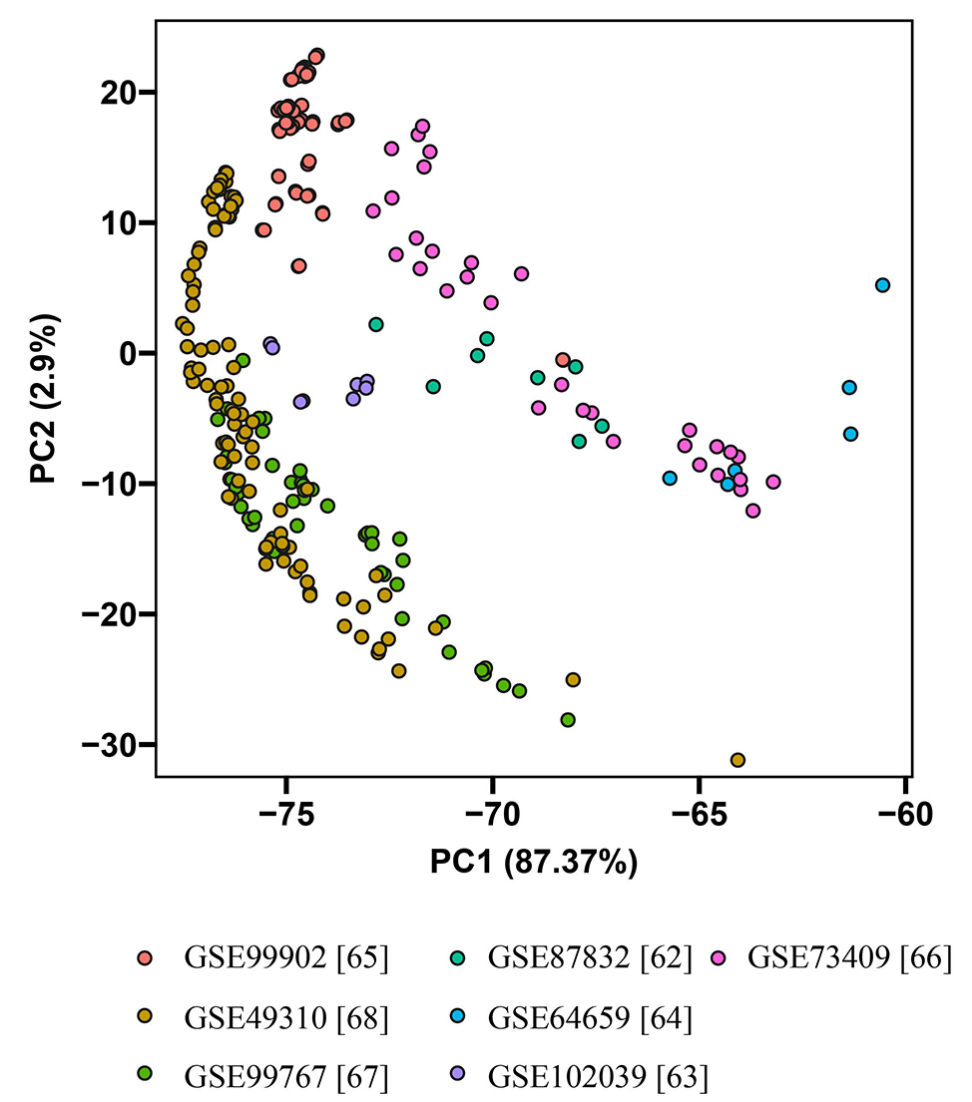
Principal Component Analysis (PCA) of RNA-seq based expression data from seven studies with 46 different conditions. The log2-transformed gene counts were used for PCA analysis

The principal component analysis (PCA) was performed on the gene counts from these seven RNA-seq studies encompassing 46 different conditions (Fig. 2). The aim of the analysis was to identify patterns and relationships among the conditions based on their gene expression profiles. The results of the PCA revealed distinct clustering patterns among the conditions from each study. Specifically, conditions within the same study tended to cluster together, indicating similarity in gene expression profiles.

Among the studies, conditions from GSE99767 [77] and GSE49310 [78] datasets exhibited close proximity and almost clustered together. Both studies, conducted by the same research group, investigated responses to acidic conditions and weak acid environments. Similarly, conditions from GSE87832 [79] and GSE73409 [80] datasets were also found to cluster close to each other. Both studies explored responses to specific treatments (casamino acids, glutamate, ⍺-ketoglutarate, and hydrogen peroxide), which suggests that there may share biological processes or pathways activated in response to these treatments. Furthermore, conditions from GSE73409 [80] and GSE64659 [81] datasets also clustered together. Both studies examined responses to different treatments (hydrogen peroxide and N-Acetylglucosamine (GlcNAc)), indicating potential shared regulatory mechanisms in response to these stressors. On the other hand, conditions from GSE99902 [82] and GSE102039 [83] datasets appeared to be unique and distinct from the other five experiments. Azadmanesh et al. [82] focused on the impact of various growth media, while Tao et al. [83] investigated responses to different air and CO_2_ environments. These distinct experimental conditions likely led to divergent gene expression patterns, indicating that these studies had different gene expression profiles compared to the other studies included in the analysis.

Overall, the PCA analysis of the gene counts from the seven RNA sequencing studies revealed clear clustering patterns, with conditions from the same study tending to group together. These findings provide valuable insights into the relationships and similarities among the different experimental conditions investigated in the RNA sequencing studies.

### Differential gene expression analysis for individual studies

Differential expression analysis was performed using DESeq2 to identify genes that exhibited significant up- or down-regulation in *C. albicans* samples under different conditions for each study, respectively (Table 2). The details of the specific comparisons, including the conditions, fold changes, and statistical significance, are provided in Additional Table 2.

**Table 2:** Summary of differential gene expression analysis and enriched GO terms. The total number of differentially expressed genes (DEGs) identified across various comparisons were shown. The differential expressed genes were established using a significance threshold of FDR-adjusted p-values along with log2 fold change criteria. Additionally, the table includes the number of significantly enriched Gene Ontology (GO) terms for each comparison

Study	Condition 1	Condition 2	No. of DEGs	No. of up regulated genes	No. of down regulated genes	No. of significantly enriched GO terms
[79]	⍺-Ketoglutarate	Glucose	1363	862	501	70
	Casamino_acid	Glucose	1413	843	570	97
	Glutamate	Glucose	1495	960	535	73
[83]	*sfl2Δ*_air	WT_air	518	336	182	0
	WT_CO_2_	WT_air	117	29	88	4
	*sfl2Δ*_CO_2_	WT_CO_2_	767	510	257	27
	*sfl2Δ*_CO_2_	*sfl2Δ*_air	6	3	3	0
[81]	GlcNAc-24 h	GlcNAc-5 h	711	340	371	243
[82]	FBS_liquid	solid	699	337	362	195
	Lees_liquid	solid	1474	793	681	232
	RMPI_liquid	solid	594	244	350	93
	YPD_liquid	solid	164	49	115	14
	Spider_liquid	solid	256	213	43	46
[80]	*rtt109 Δ* _Treated	*rtt109 Δ* _Untreated	1331	691	640	333
	*cac2Δ Δ* _Treated	*cac2Δ*_Untreated	1845	941	904	352
	*hat1Δ*_Treated	*hat1Δ*_Untreated	1412	728	684	326
	*rtt109 Δ* _Treated	WT_Treated	1201	711	490	131
	*cac2Δ*_Treated	WT_Treated	951	655	296	159
	*hat1Δ*_Treated	WT_Treated	1406	988	418	127
	*rtt109*_Untreated	WT_Untreated	375	330	45	23
	*cac2Δ*_Untreated	WT_Untreated	527	507	20	9
	*hat1Δ*_Untreated	WT_Untreated	1037	800	237	96
	WT_Treated	WT_Untreated	3061	1561	1500	357
[77]	*mig1Δ*_YPD_Acetic acid	*mig1Δ*_YPD_Untreated	2229	1301	928	193
	*mig1Δ*_YPD_Acetic acid	WT_YPD_Acetic acid	1254	874	380	83
	*mig1Δ*_YPD_Butyric acid	*mig1Δ*_YPD_Untreated	1066	628	438	70
	*mig1Δ*_YPD_Butyric acid	WT_YPD_Butyric acid	117	105	12	7
	*mig1Δ*_YPD_Untreated	WT_YPD_Untreated	178	127	51	4
	*mig1Δ*_YPM_Acetic acid	*mig1Δ*_YPM_Untreated	2481	1428	1053	228
	*mig1Δ*_YPM_Acetic acid	WT_YPM_Acetic acid	398	350	48	31
	*mig1Δ*_YPM_Butyric acid	*mig1Δ*_YPM_Untreated	2107	1099	1008	152
	*mig1Δ*_YPM_Butyric acid	WT_YPM_Butyric acid	28	14	14	3
	*mig1Δ*_YPM_Untreated	WT_YPM_Untreated	87	56	31	0
	WT_YPD_Acetic acid	WT_YPD_Untreated	1385	815	570	347
	WT_YPD_Butyric acid	WT_YPD_Untreated	974	584	390	308
	WT_YPM_Acetic acid	WT_YPM_Untreated	2640	1409	1231	189
	WT_YPM_Butyric acid	WT_YPM_Untreated	2636	1404	1232	193
[78]	Acetic acid_T1	Untreated_T1	0	0	0	0
	Acetic acid_T2	Untreated_T2	0	0	0	0
	Acetic acid_T3	Untreated_T3	155	3	152	15
	Acetic acid_T4	Untreated_T4	0	0	0	0
	Butyric acid_T1	Untreated_T1	0	0	0	0
	Butyric acid_T2	Untreated_T2	0	0	0	0
	Butyric acid_T3	Untreated_T3	1644	578	1066	177
	Butyric acid_T4	Untreated_T4	0	0	0	0
	HCl_T1	Untreated_T1	0	0	0	0
	HCl_T2	Untreated_T2	381	363	18	7
	HCl_T3	Untreated_T3	97	6	91	39
	HCl_T4	Untreated_T4	0	0	0	0
	Lactic acid_T1	Untreated_T1	106	106	0	0
	Lactic acid_T2	Untreated_T2	0	0	0	0
	Lactic acid_T3	Untreated_T3	52	2	50	15
	Lactic acid_T4	Untreated_T4	0	0	0	0
	Propionic acid_T1	Untreated_T1	3	3	0	1
	Propionic acid_T2	Untreated_T2	0	0	0	0
	Propionic acid_T3	Untreated_T3	22	3	19	0
	Propionic acid_T4	Untreated_T4	0	0	0	0

**Fig. 3:**
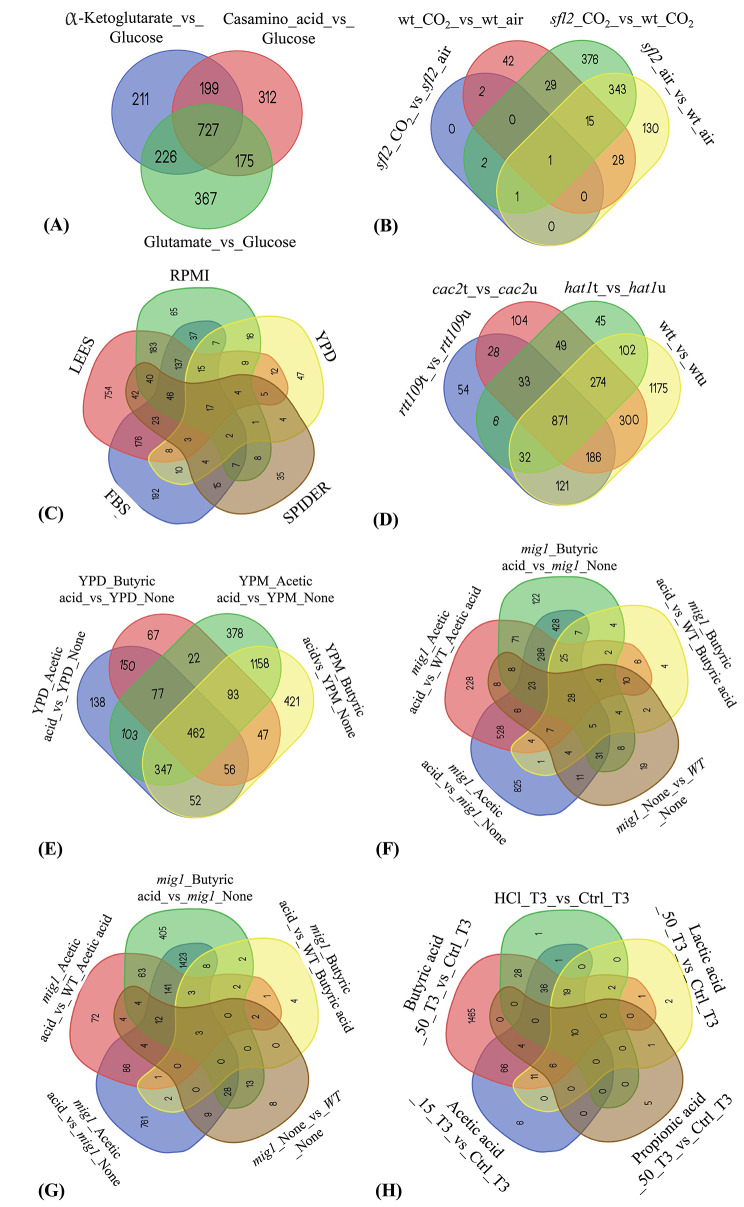
Venn diagrams illustrating shared differentially expressed genes (DEGs) across conditions within individual studies. Each Venn diagram within the figure corresponds to a specific study (**(A)**: GSE87832 [79], **(B)**: GSE102039 [83], **(C)**: GSE99902 [82], **(D)**: GSE73409 [80], **(E)**: GSE99767 [77] for the WT strain, **(F)**: GSE99767 [77] for the *mig1 d*eletion strain grown in YPD medium, **(G)**: GSE99767 [77] for the *mig1* deletion strain grown in YPM medium, **(H)**: GSE49310 [78]). Circles within each diagram represent different experimental conditions from the respective study. Overlapping regions showcase the DEGs that are shared between these conditions. The size of the overlapping regions indicates the extent of gene overlap among diverse conditions within each study

In this comprehensive analysis, we investigated the differential gene expression patterns in *C. albicans* across seven distinct studies employing DESeq2 comparisons. These studies encompassed a wide array of growth conditions, providing a diverse set of experimental settings for transcriptomic profiling. Figure 3 portrays a series of Venn diagrams, each pertaining to a specific study among the seven selected RNA-seq investigations.

Danhof et al. [79] examined the response *C. albicans* strain *SC5314* to various substrates, including glucose, casamino acids, glutamate, and α -ketoglutarate, over a five-hour period. A total of 4217 DEGs were identified. Among these, 727 genes exhibited overlapping expression profiles across the three comparisons in this study. Tao et al. [83] delved into the total RNA profiles of both wild-type and *SFL2* deletion strains under different environmental conditions, including air and a 5% CO_2_ atmosphere, over a span of 22 h. 1408 DEGs were identified and only one gene was differentially expressed in all comparisons.

Similarly, Du et al. [81] exposed *C. albicans SC5314* cells to GlcNAc for either 5 or 24 h and identified 711 genes that were differentially expressed. Azadmanesh et al. [82] examined cells grown in various liquid and solid media conditions, including FBS, LEE, RMPI, YPD, and Spider. 3187 DEGs were identified. Notably, 17 genes demonstrated overlapping expression patterns, including the noteworthy gene *SOD5* associated with oxidative stress response. Tscherner et al. [80] conducted a transcriptome analysis of wild type, *hat1∆, cac2∆, and rtt109∆* deletion strains both before and after treatment with hydrogen peroxide. A total of 13,146 DEGs were identified, and of these, 871 genes demonstrated overlapping expression profiles.

Finally, Cottier et al. [77] performed a multifaceted exploration involving two strains (*SC5314* and *mig1∆*), two media types (YPD and YPMaltose), and three different acid conditions (no acid, acetic acid, butyric acid). Similarly, Cottier et al. [78] employed a robust experimental design, analysing the transcriptional profiles of wild-type *C. albicans SC5314* under six distinct conditions, comprising both control and weak acid environments. This investigation included multiple time points and replicates, allowing for a thorough exploration of the genetic landscape in response to different weak organic acids. A total of 1970 DEGs were identified, and among them, 10 genes demonstrated overlapping expression patterns across the various weak acid environments. Notably, *CZF1, CTA4*, and *ZCF39*, genes associated with zinc homeostasis, were among the overlapping DEGs, adding further significance to these findings. These diverse experimental setups provide a rich resource for understanding the transcriptional responses of *C. albicans* under various physiological conditions.

### GO enrichment analysis

To gain further insights into the functional implications of these differentially expressed genes, GO enrichment analysis was performed using the topGo package [68], with the significantly up-regulated and down-regulated GO terms separately. Terms with a node size of > = 5 were included, and the significance of over-represented terms was assessed using the Fisher’s exact test with a threshold of FDR < 0.01. A total of 2,496 significantly up-regulated GO terms and 2,573 significantly down-regulated GO terms were identified. Among the up-regulated GO terms, 907 GO term IDs were found to be unique (after removal of duplications), indicating distinct biological processes associated with these genes. Similarly, for the down-regulated GO terms, 812 GO term IDs were identified as unique (after removal of duplications), representing specific functional categories associated with the down-regulated genes.

**Fig. 4:**
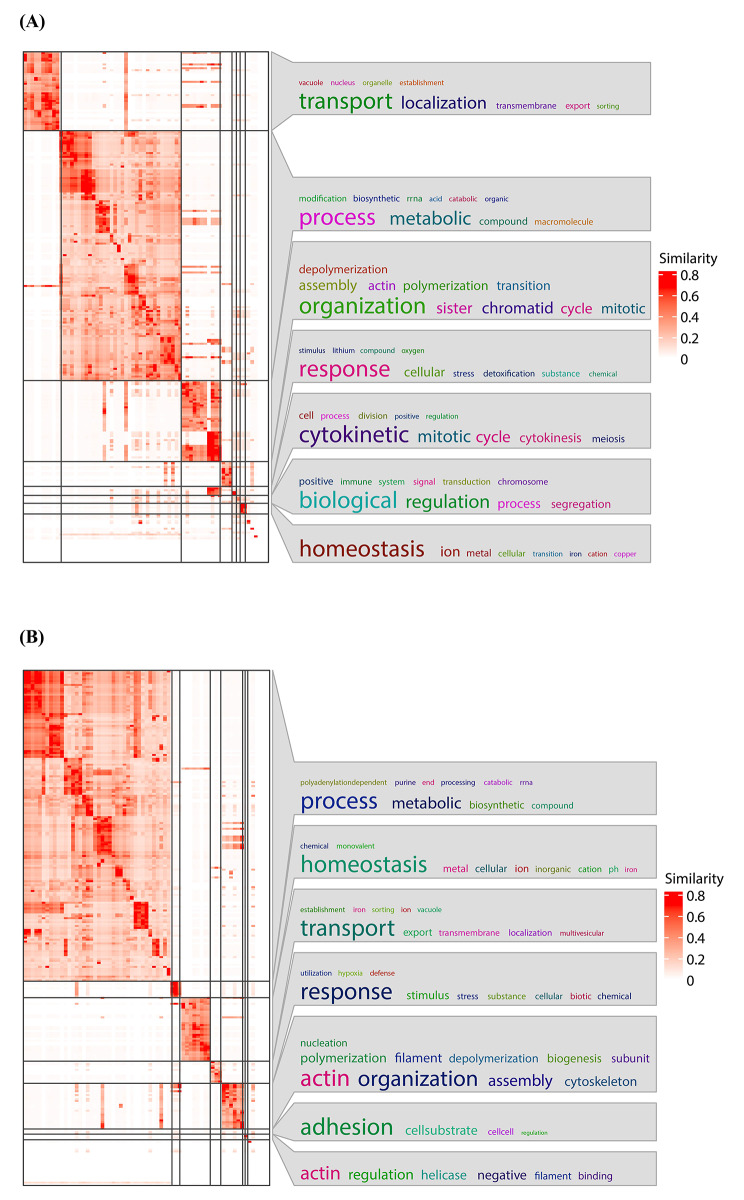
Heatmap of Clustering Patterns for Up and Down-Regulated GO Terms. To visualize the clustering patterns and relationships among the enriched GO terms, the heatmap was generated using the simplifyEnrichment method. The up-regulated GO terms were clustered into 69 binary-cut clusters **(A).** The down-regulated GO terms were clustered into 68 binary-cut clusters **(B)**

As shown in Fig. 4, the simplifyEnrichment method [84] was then applied to cluster the significantly up-regulated and down-regulated GO terms based on the semantic similarity, which facilitated the identification of distinct functional modules or pathways within the enriched GO terms. As a result, 69 distinct clusters were identified for the up-regulated GO terms, and 68 distinct clusters were found for the down-regulated GO terms. We chose the top 4 clusters with the highest GO ID numbers for further analysis, as displayed in Table 3.

**Table 3:** Overview of Selected Clusters and Main Keywords Derived from Significantly Up- and Down-Regulated GO Terms

	Cluster ID	No. of GO term IDs	Main Keywords
Up regulated GO term IDs	3	444	Process
	4	144	Organization
	1	140	Transport
	9	44	Response
Down regulated GO term IDs	1	490	Process
	3	100	Transport
	15	72	Organization
	5	35	Response

### Analysis of target gene lists in relation to up- and down-regulated GO clusters

Based on previous studies, three target gene lists related to potential antifungal targets in *C. albicans* were compiled, which can be categorized into three functional categories: central metabolism, ion homeostasis, and pathogenicity. The first gene list, comprising 24 genes associated with central metabolism, was derived from the review paper by Wijnants et al. [51]. Additionally, genes related to the glycerol pathway were incorporated into this list. The second gene list, containing 17 genes involved in ion homeostasis, was extracted from the study by Li et al. [85]. Finally, the third gene list consisted of 27 genes associated with pathogenicity, as reported by Ahmed et al. [86].

To evaluate the relevance of these target genes within the identified GO clusters, their frequency within the up and down-regulated GO term clusters was determined using g:Convert at g:Profiler. As summarized in Table 4, it showed that the specific genes in these three compiled gene lists exhibited a higher occurrence within each cluster, indicating their potential importance as antifungal targets. The complete target genes list and their frequency in the selected clusters are provided in Additiona Table 3.

**Table 4:** Frequencies of Potential Antifungal Target Genes within GO Clusters in *C. albicans*

	Gene	Frequency UP	Frequency Down	Gene Description
**Central Metabolism**	*TPS2*	4	4	Glyco_transf_20 domain-containing protein
	*TPS1*	3	3	⍺,⍺-trehalose-phosphate synthase
**Ion homeostasis**	*RIM21*	4	4	pH-response regulator protein
	*RIM9*	3	3	pH-response regulator protein
	*PRA1*	4	4	pH-regulated antigen *PRA1*
	*ZRT1*	3	3	Putative zinc transporter
	*CSR1*	3	3	Transcription factor; role in zinc homeostasis
	*MID1*	4	4	Mid1p
	*CCH1*	3	3	EF-hand domain-containing protein
**Pathogenicity**	*CPH1*	4	4	Transcription factor *CPH1*
	*SAP4*	4	4	Candidapepsin-4
	*SAP6*	4	4	Candidapepsin-6
	*SAP5*	3	3	Candidapepsin-5
	*SAP9*	3	3	Candidapepsin-9
	*SAP10*	3	3	Candidapepsin-10

Within the central metabolism category, the genes *TPS2* and *TPS1* were found to have a frequency of 4 and 3, respectively, among the up-regulated GO clusters. Similarly, these genes exhibited the same frequency within the down-regulated GO clusters. In *C. albicans*, the *TPS1* gene encodes trehalose-6-phosphate synthase (Tps1), a crucial enzyme responsible for initiating trehalose biosynthesis. Disruption of the *TPS1* gene results in reduced cell viability during oxidative stress, defective hyphal transition at 37 °C, and decreased infectivity [87–91]. While trehalose-6P phosphatase (Tps2) is the enzyme that converts trehalose-6P into trehalose, and contributes to *C. albicans’* cell viability, virulence, and susceptibility to macrophage phagocytosis [90, 92–94].

For the ion homeostasis category, several genes showed notable occurrence in both up and down-regulated GO clusters. *RIM21, RIM9, PRA1, ZRT1*, *CSR1*, *MID1*, and *CCH1* were among the genes with the highest frequencies in both categories. *PRA1, ZRT1*, and *CSR1* genes are involved in Zinc homeostasis. Disruption of *ZRT1* leads to growth defects in zinc-limited environments [95]. *PRA1* and *ZRT1* are upregulated during infection to facilitate zinc uptake [96]. Pra1 works through its ability to scavenge host zinc and it is also involved in endothelial damage, as its deletion results in shorter hyphae formation [95].

Csr1 plays a role in proliferation, hyphae formation, and biofilm maturation [97]. Deletion of *CSR1* inhibits the expression of the hypha-related gene *HWP1*, that also plays an important role during biofilm formation and for interaction with host cells [97–100]. Cch1 and Mid1 function within the Ca^2+^ cell survival (CCS) pathway in *C. albicans*, playing crucial roles in the fungus’s viability and virulence, with mutants lacking these proteins displaying decreased virulence and heightened sensitivity to azoles [101–103].

In the pathogenicity category, genes *CPH1, SAP4, SAP6, SAP5, SAP9*, and *SAP10* were identified with higher occurrence within both up and down-regulated GO clusters. *SAP4, SAP6, SAP5, SAP*9, and *SAP10* encode secreted aspartyl proteases, which are one of the three primary extracellular hydrolytic enzymes secreted by *Candida* species and the most clinically relevant [104]. Sap enzymes play a vital role in various biological activities, including hyphae formation and adherence [105, 106]. The transcription factor Cph1 plays a role in various cellular processes, including phenotypic white-opaque switching for mating and filament formation [107]. Cph1 is known to regulate genes involved in cell wall construction, such as chitin synthase genes and those related to hyphal development [108, 109].

These results highlight the potential relevance of the target genes in the context of the identified GO clusters, emphasizing their potential as important antifungal targets in *C. albicans*.

### Frequency analysis of genes in up- and down-regulated GO clusters

To gain further insights into the functional relevance of the identified up- and down-regulated Gene Ontology (GO) clusters in *C. albicans*, we conducted a frequency analysis to determine the most frequently occurring genes within these clusters. In Table 5, we present the highest ranked genes identified within the up-regulated GO clusters. Notably, the E3 ubiquitin-protein ligase gene (*RSP5*) appears in two clusters (3 and 1) with frequencies of 97 and 41, respectively. In *S. cerevisiae*, Rsp5 functions in the ubiquitin-dependent endocytosis of plasma membrane proteins [110]. The exact physiological function of Rsp5 in *C. albicans* is still not fully understood. To fully comprehend the role and significance of Rsp5 in *C. albicans*, further research is required [111].

**Table 5:** Frequently occurred genes in Up-regulated GO clusters

UP Cluster	Gene	Description	Frequency
3	*RSP5*	E3 ubiquitin-protein ligase	97
1	*RSP5*	E3 ubiquitin-protein ligase	41
4	*GLC7*	Serine/threonine-protein phosphatase	56
9	*SOD2*	Superoxide dismutase	27
9	*SOD5*	Cell surface Cu-only superoxide dismutase 5	25
9	*SOD1*	Superoxide dismutase	24
9	*SOD6*	Cell surface superoxide dismutase	23
9	*SOD4*	Cell surface superoxide dismutase	23
9	*SOD3*	Superoxide dismutase	23

Additionally, the Serine/threonine-protein phosphatase gene, *GLC7*, and the Superoxide dismutase genes *SOD2, SOD5, SOD1, SOD6, SOD4*, and *SOD3* demonstrate high occurrence in the up-regulated clusters.

Table 6 displays the genes with the highest frequency identified within the down-regulated GO clusters. These genes, along with their descriptions and frequencies, highlight the most frequently occurring genes associated with down-regulated biological processes. The ATP-dependent RNA helicase gene (*C7_03400C_A*) exhibits the highest frequency with 103 occurrences in the down-regulated clusters.

**Table 6:** Frequently occurred Genes in Down-Regulated GO Clusters

DOWN cluster	Gene	Description	Frequency
1	*C7_03400C_A*	ATP-dependent RNA helicase	103
3	*C3_06710W_A*	Not annotated	37
5	*RIM101*	pH-response transcription factor pacC/*RIM101*	15
15	*C4_01950W_A*	F-actin-capping protein subunit beta	41

Other identified genes include *C3_06710W_A* (not annotated), *RIM101* (pH-response transcription factor), and *C4_01950W_A* (F-actin-capping protein subunit) with frequencies of 37, 15, and 41, respectively. *C7_03400C_A* is the ortholog of *S. cerevisiae MTR4*, a gene encoding an ATP-dependent RNA helicase that plays a role in RNA processing [112, 113]. According to the *Candida* Genome Database (CGD), *C3_06710W* is annotated as a protein of unknown function. However, it is regulated by Sef1, Sfu1, and Hap43, underscoring a potential role in iron homeostasis. In *S. cerevisiae, VHC1* is the ortholog of *C3_06710W*. Further research is required to determine the precise function and regulatory mechanisms of *C3_06710W/VHC1* in *Candida* species. *C4_01950W_B* is annotated as a putative F-actin-capping protein subunit beta, and it is suggested to be potentially essential as homozygous deletion strains could not be obtained using the UAU1 method. In *S. cerevisiae, CAP2* is the ortholog of *C4_01950W_B*. The F-actin-capping protein subunit beta is likely involved in regulating actin filament dynamics and cytoskeletal organization. Further studies are needed to understand the specific role of *C4_01950W_B/CAP2* in *Candida* species.

## Discussion

### *Candida albicans*: challenges and antifungal target identification

*C. albicans*, a versatile opportunistic fungal pathogen, poses significant challenges in the clinical management of candidiasis. The rise in immunocompromised patients and the widespread use of broad-spectrum antibiotics have led to an increased incidence of *C. albicans* infections [11, 20]. Understanding the pathogenicity mechanisms of *C. albicans* is pivotal for developing effective antifungal therapies and diagnostics [114].

The ability of *C. albicans* to transition between different morphological states, such as yeast and hyphal cells, or between the epigenetic white and opaque phases, contribute to its pathogenicity and ability to form biofilms of which the extracellular matrix acts as a protective shield against host defenses and antifungal treatments [26, 30, 115, 116]. Specific gene families governing adhesion, proteolysis, and defense against reactive oxygen species play significant roles depending on the infection site [8, 117]. Moreover, the metabolic flexibility of the fungus, allowing it to thrive in diverse environments contributes to reduced susceptibility to antifungals and aids in evading host defenses [118].

A promising approach in the quest for innovative and potent antifungals is the strategic targeting of fungal virulence mechanisms. Disrupting these traits holds significant potential for the advancement of novel therapeutic strategies [119, 120]. The intricate process of transcriptional regulation plays a pivotal role in these adaptive mechanisms. As such, transcriptomic studies conducted throughout the course of an infection, or in conditions that mimic this process, offer invaluable insights. Through transcriptomics analysis, we gain the ability to elucidate the essential pathways required for fungal adaptation within the host environment [114, 121, 122].

**Fig. 5:**
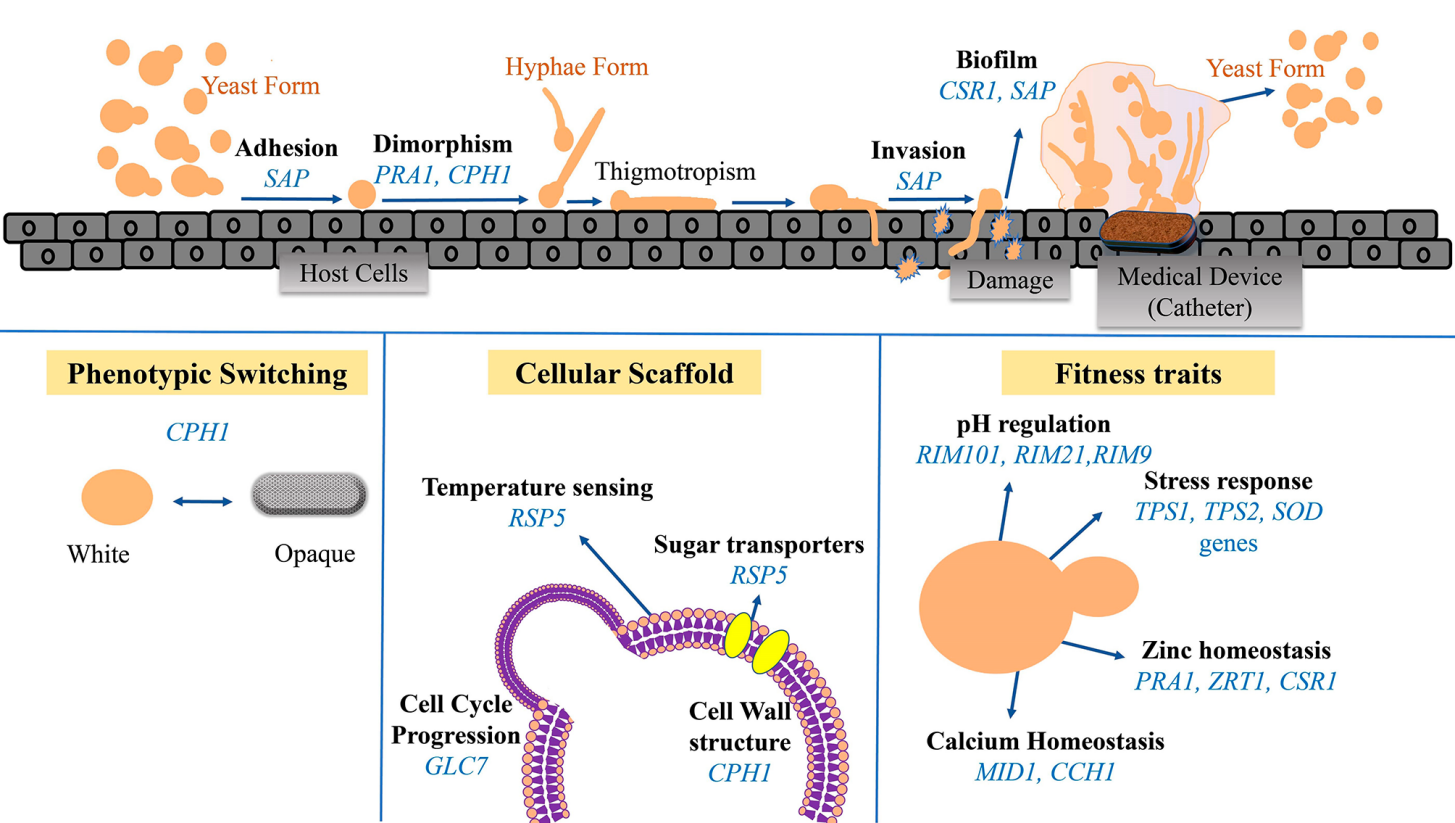
Key Genes and Cellular Processes in *C. albicans* Virulence. Key genes identified in this study play pivotal roles in critical cellular processes crucial for the virulence and survival of *C. albicans*. These processes encompass morphological transitions, including adhesion, dimorphism, hyphae formation, invasion, and biofilm formation. Additionally, the genes contribute to phenotypic switching, regulating the transition between white and opaque phases. They also form the cellular scaffold, orchestrating essential functions such as temperature sensing, sugar transport, cell wall structure, and cell cycle progression. Moreover, these genes influence fitness traits, including pH regulation, stress response, and zinc and calcium homeostasis. Their significance in these vital cellular processes underscores their potential as promising antifungal targets for combatting *C. albicans* infections

Our transcriptomic meta-analysis revealed genes with high frequency in the selected GO clusters, most of which are associated with different virulence factors, as shown in Fig. 5. Importantly, some of these genes have already been suggested as potential antifungal targets.

In our investigation, we introduced an innovative approach that utilizes the wealth of publicly accessible RNA sequencing data for a thorough meta-analysis. This method is designed to extract valuable insights from extensive transcriptomic datasets, providing a systematic analysis that complements experimental studies. By unveiling new perspectives and potential targets, this approach helps to understand complex biological systems better and offers guidance for future experimental inquiries.

### Key genes associated with central metabolism, ion homeostasis and pathogenicity

Our meta-analysis revealed several key genes that showed associations with central metabolism, Ion homeostasis and pathogenicity in *C. albicans*.

### Central metabolism and trehalose synthesis

Within the central metabolism category, the genes *TPS2* and *TPS1* were identified with high frequencies in both up and down-regulated clusters. These genes encode the primary enzymes involved in the synthesis of trehalose, a stress protectant molecule that plays a crucial role in the response to various environmental stresses [123, 124]. That these two genes clearly come out of our analysis supports our approach as they were previously already linked to virulence in a number of pathogenic fungi [90, 125–127].

The absence of Tps1 and Tps2 in mammals, coupled with their pivotal roles in cell viability and virulence, positions them as promising targets for novel antifungal medications [90, 128].

Miao et al. [129] explored the complex structural characteristics of Tps2 enzymes across different catalytic phases, elucidating the mechanisms behind substrate recognition and phosphate removal. Significantly, the similarities in both structure and function of Tps2 proteins across various pathogenic fungi, including *C. albicans*, underscore their specialization for the trehalose pathway, offering the potential for tailored enzyme inhibitors to minimize unforeseen side effects. Additionally, their findings indicate that minor alterations to Tps2’s catalytic pocket might disrupt fungal resistance to drug binding while hindering the conversion of trehalose-6-phosphate (T6P) into trehalose [129]. This disruption could potentially yield highly potent antifungal drugs by preventing the production of trehalose from T6P, consequently causing the accumulation of toxic T6P [90].

T6P itself has shown promise as an inhibitor of Tps1, paving the way for the development of T6P analogs as potential antifungal agents [128]. Similarly, the development of specific inhibitors against Tps2 can aid in the discovery of novel antifungals against *C. albicans* infections and those caused by other human fungal pathogens [90, 125–127].

### Zinc homeostasis

The genes *PRA1, ZRT1*, and *CSR1* exhibited high frequencies in both up and down-regulated clusters in the ion homeostasis category. These genes play crucial roles in regulating zinc homeostasis, which significantly impacts various aspects of *C. albicans’* biology and pathogenesis. *C. albicans* has developed mechanisms to tightly control intracellular zinc levels because of the toxicity of excess zinc [130].

Targeting zinc homeostasis shows potential for developing antifungal drugs. Zinc-attenuating compounds, such as ZAC307 and ZAC989, have been identified and demonstrated antifungal activity by chelating zinc in vitro [131]. These compounds have also shown efficacy in murine fungal infection models, indicating their potential as a novel class of antifungal agents [131].

### Calcium homeostasis

*MID1* and *CCH1* are two other genes that showed high frequency in the ion homeostasis category. In *C. albicans*, Cch1 and Mid1 are essential components of the Ca^2+^ cell survival (CCS) pathway, which regulates calcium homeostasis [102]. Disruption of this pathway can result in growth problems and cell death in eukaryotic organisms [132]. These proteins, Cch1 and Mid1, are homologous to the catalytic and regulatory subunits of mammalian voltage-gated calcium channels, respectively [133, 134].

### *SAP* genes and *CPH1*: keys to *C. albicans* pathogenicity

In the pathogenicity category, genes *SAP4, SAP6, SAP5, SAP*9, *SAP10, and CPH1* were consistently present with high frequencies in both up and down-regulated clusters. Secreted Aspartyl Proteases (SAPs) are crucial for *C. albicans* to establish infections. They enable the fungus to degrade host tissues, adhere to mucosal surfaces, and form biofilms, all of which contribute to their pathogenicity [135–137]. Recent research by Dhanasekaran, et al. [138] explored the potential of bioactive components from medicinal herbs as inhibitors of Sap enzymes. The study identified hesperidin and vitexin as promising candidates based on their drug-likeness, safety, and their ability to interact with the catalytic site of the Sap5 enzyme.

Recent research by Wagner et al. [139] revealed that the transcription factor Cph1 plays a key role in the unmasking of immunogenic elements, such as ß (1,3)-glucan and chitin, in *C. albicans*. This unmasking process mediated by Cph1 triggers immune responses, including the activation of immune cells like macrophages and neutrophils, ultimately leading to enhanced fungal clearance during infection [139]. Understanding the mechanisms underlying Cph1-mediated unmasking provides valuable insights into host-fungus interactions and potential therapeutic strategies for combating *C. albicans* infections.

### Exploring potential antifungal targets in *C. albicans*

Through frequency analysis, several key genes were found to be frequently occurring in the up/down regulated clusters, which include *RSP5, GLC7, SOD2, SOD5, SOD1, SOD6, SOD4, SOD3, C7_03400C_A, RIM101, C3_06710W* and *C4_01950W_A*.

### RSP5 and GLC7

Rsp5, an E3 ubiquitin ligase, is a central player in temperature sensing, coordination of the heat shock response, and regulation of sugar transporters, all of which significantly impact *Candida’s* pathogenicity and its ability to adapt to different environments, as indicated by previous studies [111, 140]. However, the precise physiological functions of Rsp5 in *C. albicans* remain incompletely understood and further research is necessary to gain a comprehensive understanding of its role and its significance [111].

*GLC7*, the gene encoding the Serine/threonine-protein phosphatase Glc7, functions in various cellular processes in *C. albicans*, including modulating cell morphology, regulating cell cycle progression, facilitating DNA damage response, and enhancing stress resistance [141, 142].

### Role of *SOD* genes

The *SOD* gene family in *C. albicans* plays a key role in protecting the fungus against oxidative stress, enabling its survival within the host environment [143].

Bink et al. [144] reported that the efficacy of miconazole against *C. albicans* biofilms can be improved by utilizing Sod inhibitors. These results underscore the important role played by Sod’s in the formation of miconazole-tolerant persister cells in *C. albicans* biofilms, primarily through their ROS detoxifying activity. Earlier studies have also implicated Sod4 and Sod5 in ROS detoxification in *C. albicans* [145, 146]. The active-site structure and copper binding characteristics of Sod5, a copper-only enzyme, significantly differ from those of Cu/Zn-SODs found in animal hosts. These distinctions highlight the potential of targeting Cu-only Sod*’s* as a viable approach for future antifungal drug design [147, 148].

### Targeting the Rim101 pH response pathway

Activation of the Rim101 pathway leads to the expression of specific genes involved in various cellular processes and virulence factors, including growth, iron metabolism, cell wall structure, yeast-to-hypha transition, adhesion, and biofilm formation [149]. Disruption of the *RIM101* gene in *C. albicans* leads to increased susceptibility to both echinocandins and azoles, indicating the involvement of the Rim pathway in tolerance and/or resistance to these antifungal drugs [149–151].

Targeting the Rim101 pH response pathway, which is specific to fungi and conserved among fungal species, holds promise for the development of novel antifungal strategies. Combining the targeting of the Rim pathway with existing antifungal drugs may represent a potent approach in combating *C. albicans* infections [149–151].

### Incorporating the findings of the meta-analysis in relation to the original studies

Our meta-analysis uncovered several areas of agreement and divergence between our findings and the original studies, shedding light on the intricate regulatory mechanisms governing *C. albicans’* response to specific stressors.

In the study conducted by Cottier et al. [77], the investigation centered around the genetic and molecular responses of *C. albicans* to acetic and butyric acids, with an emphasis on the influence of glucose availability and the *MIG1* gene on weak organic acid (WOA) sensitivity. The study identified a set of six genes, including *GLG2, ALD6, FDH1*, and *HGT16*, consistently upregulated under conditions of heightened WOA sensitivity. Intriguingly, our meta-analysis did not highlight any of these genes as significant in the context of *C. albicans* stress responses. This discrepancy suggests that while these genes play a role in WOA sensitivity, their contribution might not be as universal as initially proposed. Similarly, in Cottier et al’s study [78], the investigation delved into the responses of *C. albicans* to WOAs produced by bacteria present in the human host. The study identified a core transcriptional response encompassing genes associated with iron homeostasis, carboxylic acid metabolism, and ribosome biogenesis. However, none of these genes emerged as prominent candidates in our meta-analysis, indicating that the regulatory pathways governing WOA responses might vary across different experimental conditions.

Our meta-analysis also connected findings from Danhof et al. [79], who explored genetic factors influencing medium alkalinization during growth on ⍺-ketoglutarate, with those of Cottier et al. [77]. The study highlighted the role of the *CPH1* gene in this process. Interestingly, our analysis supported this finding, particularly in dataset GSE99767 [77], where variations in carbon sources led to the upregulation of *CPH1*. This convergence of results underlines the robustness of the *CPH1* gene’s involvement in *C. albicans’* responses to specific stressors.

Furthermore, Tscherner et al. [80] focused on the role of histone acetyltransferases (HATs) in oxidative stress resistance. Although the study revealed a set of oxidative stress-related genes, our meta-analysis did not identify these specific genes as central components of *C. albicans’* stress response network. However, the study highlighted *SOD* genes, particularly Sod4 and Sod5, as essential for survival upon phagocytosis. Interestingly, our meta-analysis did identify these *SOD* genes as potential antifungal targets, reinforcing their significance in *C. albicans’* interactions with the host immune system.

In conclusion, our meta-analysis provides a comprehensive overview of the transcriptional responses of *C. albicans* to various stressors. While there are instances of agreement with the original studies, such as the role of *CPH1* and *SOD* genes, there are also instances of non-convergence, as seen with genes identified in [77] and [78]. These disparities emphasize the context-dependent nature of *C. albicans’* responses to stressors and highlight the need for a nuanced understanding of the regulatory mechanisms governing these responses. Our findings not only contribute to deciphering the intricate stress response network of *C. albicans* but also provide insights into potential avenues for future research into antifungal strategies targeting specific stress-responsive genes.

### Limitations and future directions

The findings from our meta-analysis highlight the potential of these identified genes as targets for the development of novel antifungal therapies against *C. albicans*. By focusing on key genes involved in central metabolism, ion homeostasis, and pathogenicity, we can potentially disrupt critical cellular processes and enhance the efficacy of antifungal treatments.

Moreover, the meta-analysis approach employed in this study demonstrates the power and benefits of integrating and analyzing large-scale publicly available transcriptomic data. As expected, variations in experimental conditions, sample sizes, and data preprocessing methods across different studies can introduce potential biases and heterogeneity. In this study, the RNA-seq data were analyzed with a unified bioinformatics pipeline, which reduced the biases introduced by software.

With gene expression data from different conditions, it provides a comprehensive overview of the transcriptomic landscape of *C. albicans*, highlighting the interconnection of different biological processes and pathways. By examining the collective gene expression changes across multiple studies, we can gain a more holistic understanding of *C. albicans’* response to stress, its adaptation mechanisms, and its virulence factors. This information can guide future research efforts and aid in the development of targeted interventions. It is important to acknowledge the limitations of our study. While meta-analysis provides valuable insights, it relies on the availability and quality of publicly accessible transcriptomic data. Therefore, cautious interpretation of the results is necessary, and additional experimental validations are needed to confirm the functional significance of the identified target genes.

## Conclusion

In conclusion, our meta-analysis of transcriptomic data in *C. albicans* revealed key genes associated with antifungal resistance and pathogenicity. Genes involved in central metabolism, ion homeostasis, and pathogenicity emerged as potential targets for therapeutic interventions. The findings underscore the importance of integrated data analysis approaches and highlight the benefits of meta-analysis in uncovering novel insights and potential therapeutic avenues. Further research and experimental validation are warranted to elucidate the precise roles of these genes and their potential as targets for antifungal strategies.

### Electronic supplementary material

Below is the link to the electronic supplementary material.

Supplementary Material 1. Additional Table .xlsx contains detailed samples information

Supplementary Material 2. Additional Table .xlsx contains DESeq2 detailed results

Supplementary Material 3. Additional Table .xlsx contains the complete target gene lists and their frequency in the selected GO clusters

## Data Availability

Not applicable.

## Abbreviations

CCS: Ca^2 +^ cell survival
CGD: *Candida* genome Database
DEGs: Differentially expressed genes
ENA: European Nucleotide Archive
FDR: False Discovery Rate
GEO: Gene Expression Omnibus
GO: Gene Ontology
HATs: Histone acetyltransferases
GlcNAc: N-Acetylglucosamine
PCC: Pearson correlation coefficient
PCA: Principal component analysis
QC: Quality control
SAPs: Secreted aspartyl proteases
SOD: Superoxide dismutase
TPM: Transcripts per million
T6P: Trehalose-6-phosphate
Tps: Trehalose-6-phosphate synthase
Tps2: Trehalose-6-phosphate phosphatase
WOA: Weak Organic Acid
